# 

**Published:** 1898-12-10

**Authors:** 


					the HOSPITAL. " The standard of highest purity."?LancetDeo. 16th, 1898.
CADBURY'S TJf to* CADBURY'S
OOOOA *, JEH OOOOA
ABSOLUTELY PURE, NO DRUGS OR ALKALIES USED.
Am*
OP WICH Hnsri Ta I
Wo. 6^7. Vol XXV. ?g?S3S0 Saturday,
Dec. 10th, 1898.
[SubscriptionJTerma,] paICB TWOPENCE.iT

				

